# Refining Biodegradability Assessments of Polymers Through Microbial Biomolecule Quantification

**DOI:** 10.3390/polym17172376

**Published:** 2025-08-31

**Authors:** Woo Yeon Cho, Pyung Cheon Lee

**Affiliations:** 1 Molecular Science & Technology Research Center (MSTRC), Ajou University, Woncheon-dong, Yeongtong-gu, Suwon 16499, Republic of Korea; wycho418@ajou.ac.kr; 2 Advanced College of Bio-Convergence Engineering, Ajou University, Woncheon-dong, Yeongtong-gu, Suwon 16499, Republic of Korea

**Keywords:** biodegradable polymers, biodegradability assessment, biochemical assays, microbial dynamics, soil ecosystems

## Abstract

The accumulation of plastic waste has intensified the pursuit of biodegradable alternatives, yet standard methods such as CO_2_ evolution, oxygen demand, and mass loss fail to fully capture microbial physiological responses during degradation. This study introduces a biochemical assay-based approach to quantify proteins, lipids, and carbohydrates in soil as indicators of microbial activity during polymer biodegradation. For microcrystalline cellulose (MCC), proteins, lipids, and carbohydrates increased by 2.09-, 6.47-, and 11.22-fold, respectively (all *p*-values < 0.001), closely aligning with CO_2_ evolution trends. Non-biodegradable poly(vinyl chloride) (PVC) exhibited no significant changes. Synthesized poly(butylene glutarate) (PBG) also showed significant biomolecule accumulation (up to 2.70-fold) alongside CO_2_ production. Biomolecule quantification complements CO_2_-based methods by revealing microbial proliferation and metabolic activity that persist beyond the mineralization plateau, offering a more comprehensive assessment of biodegradability.

## 1. Introduction

Plastic pollution is a pressing global challenge with profound ecological and human health consequences [1]. To address this issue, biodegradable plastics have been developed as alternatives to conventional polymers, offering potential solutions for reducing persistent plastic waste [2]. Over the past decade, global plastic production has increased from approximately 381 million tons in 2015 to about 435 million tons in 2020, with waste generation following a similar trajectory. In 2019 alone, plastic waste reached approximately 353 million tons—over double the amount recorded in 2000. Projections by the OECD indicate that plastic production and waste could increase by 70% by 2040 and nearly triple by 2060, with less than 12% being recycled and most destined for landfill or environmental release [3,4,5]. Ensuring effective biodegradation of these materials across diverse environments remains a critical scientific and regulatory concern [6], and research is increasingly focused on enhancing both biodegradability and mechanical performance [7].

Plastic biodegradation is a microbially mediated process in which polymers are broken down into lower-molecular-weight compounds and ultimately mineralized into carbon dioxide (CO_2_), methane (CH_4_, under anaerobic conditions), water, and biomass [8,9]. This process occurs through enzymatic hydrolysis, oxidation, or depolymerization, followed by microbial assimilation of the degradation products into central metabolic pathways. Microbial activity is thus a key driver of polymer degradation in soils, marine systems, and composting environments, making its assessment essential for a comprehensive understanding of biodegradability [10].

Conventional biodegradability tests—such as CO_2_ evolution [11,12], oxygen demand [13,14], and mass loss measurements [15,16]—are valuable for tracking mineralization and disintegration but do not directly capture microbial metabolic activity. The frequent conflation of biodegradation with physical disintegration can also lead to overestimations of environmental degradability [17]. Biodegradation generally proceeds through biodeterioration, biofragmentation, assimilation, and mineralization [18], with abiotic factors (e.g., hydrolysis, UV oxidation, and mechanical forces) initiating breakdown and microbial metabolism driving further decomposition [19,20]. While CO_2_ evolution and weight loss remain standard, techniques such as scanning electron microscopy (SEM) and Fourier-transform infrared spectroscopy (FTIR) provide structural and chemical insights [21]. This study focuses specifically on microbial responses, while acknowledging that abiotic processes merit future investigation.

Recent work suggests that proteins, lipids, and carbohydrates can serve as biochemical markers of microbial activity during biodegradation [22,23,24,25]. These macromolecules reflect physiological changes in microbial communities, providing complementary insights to CO_2_-based metrics. While mineralization data indicate carbon turnover, biochemical markers reveal metabolic activity at the cellular level, potentially offering earlier and more nuanced detection of biodegradation processes. Here, we present a quantitative biochemical assay-based method to measure soil macromolecule accumulation as a proxy for microbial responses during polymer degradation. We hypothesized that biodegradation would significantly increase soil protein, lipid, and carbohydrate concentrations. To test this, we compared biochemical data with CO_2_ measurements to assess the method’s validity.

Unlike colony-forming unit (CFU) counts, which capture only culturable microbes, or DNA-based approaches, which do not distinguish between active and dormant cells, biomolecular assays provide a direct measure of microbial metabolic activity. Although promising, further validation is needed to confirm their specificity as direct indicators of polymer degradation. Future studies should integrate metagenomic, metatranscriptomic, and enzymatic profiling approaches to link biochemical markers to microbial community structure and function [26]. Overall, this study aims to integrate biomolecular quantification with conventional assays to develop a more comprehensive and physiologically informed framework for assessing polymer biodegradability.

## 2. Materials and Methods

### 2.1. Materials

Poly(vinyl chloride) (PVC, high molecular weight, 81387), microcrystalline cellulose (MCC, 20 μm, 310697), glass beads (425–600 μm, G8772), anthrone (97%, 319889), and sodium citrate dihydrate (≥99%, W302600) were purchased from Sigma-Aldrich (St. Louis, MO, USA). All polymer samples were stored at room temperature (~25 °C) in their original sealed packaging until the start of the biodegradation assay to prevent pre-exposure to environmental factors such as humidity or temperature fluctuations. No additional pre-conditioning (e.g., controlled humidity exposure or thermal treatment) was performed. Sample dimensions were obtained from the manufacturer’s product data sheet and were not independently measured.

Ethyl acetate (99.9%, 000E0686), ethanol (99.9%, 000E0691), phosphoric acid (85%, 000P0458), and NaOH (>98%, 000S0610) were purchased from Samchun Chemical Co., Ltd. (Seoul, Republic of Korea). Sulfuric acid (98%, 7683-4100), CuSO_4_·5H_2_O (99%, 2588-4405), and Na_2_CO_3_ (99%, 7541-4405) were obtained from Daejung Chemical & Metals Co., Ltd. (Siheung, Republic of Korea). KCl (99%, 370) and potassium sodium tartrate (99%, 4207) were purchased from Duksan Pure Chemical Industry Ltd. (Ansan, Republic of Korea). Bovine serum albumin (BSA) standard (1 mg·mL^−1^, SK3041-1) was obtained from Bio Basic Inc. (Markham, ON, Canada). Vanillin (≥98%, sc-251423) was purchased from Santa Cruz Biotechnology, Inc. (Dallas, TX, USA), and Folin–Ciocalteu phenol reagent (96703S8130) from Junsei Chemical Co., Ltd. (Tokyo, Japan). Glucose was purchased from Samyang Corporation, Ltd. (Seoul, Republic of Korea), and soybean oil was obtained from a local market (Suwon, Republic of Korea).

Poly(butylene glutarate) (PBG) was synthesized via melt polycondensation of glutaric acid and 1,4-butanediol without an additional catalyst, relying on the carboxylic acid groups in the monomers to drive esterification and subsequent transesterification. The physicochemical properties and physical appearances of all tested polymers are presented in Table S1 and Figure S1.

A SpectraMax^®^ Plus 384 spectrophotometer (Molecular Devices, Sunnyvale, CA, USA) was used to quantify carbohydrate, lipid, and protein contents in soil samples. Organic solvents were removed using an EZ-2 Plus evaporator (Genevac Ltd., Ipswich, UK) in low-boiling-point mode. Soil samples were homogenized using a Precellys 24 homogenizer (Bertin Technologies, Montigny-le-Bretonneux, France).

### 2.2. Biodegradation Assay Setup and Conditions

MCC, PVC, and blank controls were used to quantitatively assess biodegradability via biomolecule analysis. MCC served as the positive (biodegradable) reference material, PVC as the negative control, and blank soil (without added test material) to normalize CO_2_ evolution from each test vessel.

Soil was collected from an undisturbed site at Ajou University (37°17.1210′ N, 127°2.6710′ E) to minimize anthropogenic contamination from pesticides and fertilizers. The site had not been used for recent agricultural activities, preserving native microbial communities. The soil had a mean pH of 6.88 and a mean moisture content of 39.2%, measured using a soil hygrometer (DM-5, TAKEMURA Electric Works, Tokyo, Japan).

Following ISO 17556:2019 recommendations [8], the soil was used in its natural state without sterilization to maintain indigenous microbial dynamics. It was air-dried at room temperature for 24 h, sieved through a 2 mm mesh to remove debris, and supplemented with minerals and water (Table 1) to provide suitable conditions for biodegradation testing. Although a full chemical and microbiological characterization of the soil was not performed, its suitability was confirmed through its ability to sustain microbial activity, as evidenced by CO_2_ evolution and biomolecule accumulation during the preliminary trials.

**Figure 1:**
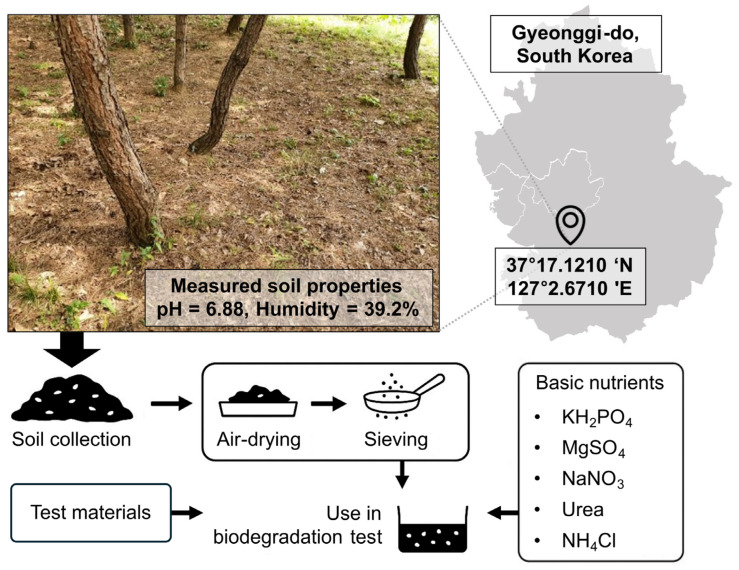
Schematic overview of the soil sampling site, measured physicochemical properties (pH and humidity), and the preparation workflow prior to biodegradation testing. Soil was collected from an undisturbed site at Ajou University (37°17.1210′ N, 127°2.6710′ E), characterized for pH and moisture content, air-dried for 24 h, sieved through a 2 mm mesh sieve, and supplemented with minerals and water before use in biodegradation assay.

Each vessel contained 10 g of test material thoroughly mixed with 500 g of soil, corresponding to 2% (*w*/*w*). Experiments were conducted under aerobic conditions in the dark at 25 °C with a continuous airflow rate of 200 mL·min^−1^. CO_2_ evolution was monitored using an infrared (IR) detector. Sampling was performed in duplicate vessels (biological replicates) at three phases: initial (T_0_, 0 days, baseline), exponential (T_1_, 10 days), and stabilization phase (T_2_, 40 days). The total experimental period was 43 days, sufficient to capture key biodegradation trends while avoiding unnecessary sampling beyond the stabilization (or plateau) phase. At each time point, quantitative measurements were conducted in triplicate per replicate vessel, resulting in six data points per material per sampling phase. The soil sampling site, measured properties, and preparation processes are summarized in Figure 1.

Biodegradability was calculated as the percentage of theoretical CO_2_ (ThCO_2_) based on the carbon content of the test sample, according to Equation (1):

ThCO_2_ = (44/12) × *m* × *w_c_*. (1)

where *m* is the test sample mass (mg), *w_c_* is the carbon content, 44 is the molecular weight of CO_2_, and 12 is the atomic weight of carbon.

Biodegradability at time t (*Dt*) was calculated using Equation (2)

*Dt* = [(∑CO_2_^T^ − ∑CO_2_^B^)/ThCO_2_] × 100 (2)

where ∑CO_2_^T^ is the cumulative CO_2_ (mg) generated by the test sample from the start of the experiment to time t, and ∑CO_2_^B^ is the cumulative CO_2_ generated by the blank sample over the same period.

### 2.3. Biodegradation Experiments for PBG

To further validate the applicability of biomolecular indicators, an additional biodegradation experiment was conducted using polybutylene glutarate (PBG) along with blank controls. Only two groups were tested: PBG and blank soil (without added material). Each test vessel contained 8 g of PBG mixed with 500 g of soil. The soil used in this experiment was a 1:1 (*w*/*w*) mixture of topsoil collected from Ajou University and from Hwaseong-si, Gyeonggi-do, Republic of Korea (37°10.8030′ N, 126°58.8720′ E; mean pH of 6.86, and mean humidity of 66.5%). The mixed soil was processed as described in Section 2.2, including air-drying, sieving through a 2 mm mesh, and supplementation with mineral and water (Table 1).

The experiment lasted 48 days, with sampling performed only at the endpoint (day 48), corresponding to the plateau phase of microbial activity. Endpoint sampling was chosen to capture cumulative microbial responses, a strategy commonly applied in long-term soil incubation studies. Three independent vessels (triplicates) were prepared for each group, and quantitative biomolecular analyses were conducted once per vessel, resulting in three data points per group. All other conditions, including incubation temperature, airflow rate, and CO_2_ monitoring methods, were identical to those in Section 2.2.

### 2.4. Biomolecular Quantification

**Figure 2:**
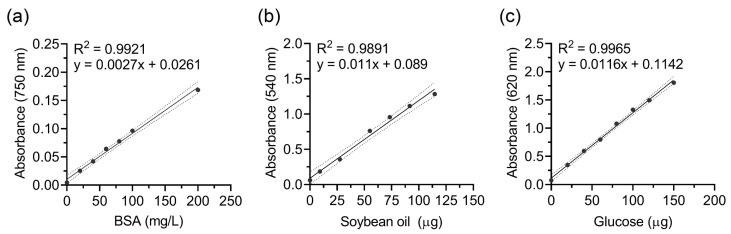
Calibration curves for quantifying (**a**) protein, (**b**) lipid, and (**c**) carbohydrate content in soil samples. Protein, lipid, and carbohydrate calibrations were performed using bovine serum albumin (BSA), soybean oil, and glucose, respectively. All calibration curves exhibit high linearity (R^2^ > 0.98), confirming the reliability of the quantitative assays.

The biochemical quantification assays were validated using calibration curves generated with bovine serum albumin (BSA), soybean oil, and glucose as standards (Figure 2). These compounds were selected as representative biomolecules for proteins, lipids, and carbohydrates, respectively, enabling robust quantification of microbial biomolecule accumulation. Colorimetric assays included the modified-Lowry method for proteins [27,28], the sulfo-phospho-vanillin (SPV) reaction for lipids [29,30], and the anthrone–sulfuric acid assay for carbohydrates [31]. These assays are widely used in soil and environmental microbiology due to their simplicity, low cost, and robustness against interference from humic substances, and thus serve as functionally relevant surrogates for microbial metabolic activity where direct biomass measurement is impractical [32]. Calibration curves were established under controlled conditions rather than by direct spiking into soil to avoid variability from interactions with organic matter and native microbial communities.

This biochemical approach was selected over DNA quantification because environmental DNA may persist from inactive or lysed cells and does not necessarily reflect microbial metabolic activity. In contrast, the accumulation of proteins, lipids, and carbohydrates provides a more direct indicator of microbial responses during biodegradation.

### 2.5. Protein Quantification

Soil protein content was determined using a modified Lowry assay [27,28], which is widely used to estimate total protein content in soil extracts and thus microbial biomass in complex environmental samples. For sample preparation, 1 g of air-dried soil was placed in a 15 mL polypropylene tube with 8 mL of 20 mM sodium citrate (pH 7.0), vortexed for 10 s, and autoclaved at 121 °C for 30 min. After cooling on ice for 10 min, 1 mL of the mixture was transferred to a 1.75 mL microtube and centrifuged at 16,000× *g* for 3 min at 4 °C. The supernatant was collected for analysis.

Reagents were prepared as follows: (1) Solution 1: 3.5 g CuSO_4_·5H_2_O in 100 mL distilled water; (2) Solution 2: 7.0 g potassium sodium tartrate in 100 mL distilled water; and (3) Solution 3: 7.0 g Na_2_CO_3_ in 100 mL of 0.35 N NaOH. Reagent α was prepared by mixing Solutions 1, 2, and 3 in a 1:1:100 volume ratio; Reagent β was prepared by mixing distilled water, Solution 2, and Solution 3 in the same ratio.

Supernatants were dispensed into two 96-well plates (plates A and B) at 20 µL per well, followed by 80 µL phosphate-buffered saline (PBS). To plate A, 100 µL of Reagent α was added; to plate B, Reagent β was added. Plates were incubated in the dark at room temperature for 10 min. Diluted Folin–Ciocalteu phenol reagent (1:10 in distilled water) was then added (100 µL per well) and incubated in the dark for 30 min at room temperature. Absorbance was measured at 750 nm, and protein content was calculated as

Protein content = 1.25 × (Abs_plate A_ − Abs_plate B_)

### 2.6. Lipid Quantification

Soil lipid content was determined using a modified SPV assay [29,30], a sensitive method compatible with organic solvent extracts from environmental samples. For extraction, 300 mg soil, 500 mg glass beads, and 1 mL of ethyl acetate-ethanol (2:1 *v*/*v*) were added to a 2 mL screw-cap microtube, homogenized at 3000× *g* for 30 s, paused for 30 s, and homogenized again. The mixture was centrifuged at 16,000× *g* for 3 min at room temperature, and 600 µL supernatant was transferred to a new tube. Next, 400 µL ethyl acetate and 200 µL 0.85% KCl were added, followed by a second homogenization and centrifugation. A 500 µL aliquot of supernatant was collected, and solvents were evaporated using an EZ-2 Plus evaporator (low BP mode). The lipid residue was incubated with 200 µL concentrated sulfuric acid at 100 °C for 10 min, cooled to 4 °C for 2 min, and then incubated in the dark at room temperature for 10 min. Vanillin solution (0.2 mg·mL^−1^ in 17% H_3_PO_4_, 500 µL) was added, vortexed, and incubated at 37 °C for 15 min, followed by 10 min at room temperature in the dark. Finally, 200 µL of each sample was transferred to a 96-well plate, and absorbance was read at 530 nm.

### 2.7. Carbohydrate Quantification

Soil carbohydrate content was determined using a modified anthrone–sulfuric acid assay [31], which is widely used due to its simplicity, reproducibility, and tolerance to humic interference. For extraction, 400 mg soil, 500 mg glass beads, and 1 mL of distilled water were added to a 2 mL screw-cap microtube and homogenized at 3000× *g* for 30 s, paused for 30 s, and homogenized again. The mixture was centrifuged at 16,000× *g* for 3 min at room temperature, and the supernatant was collected. The extraction was repeated with an additional 1 mL of water, and combined extracts were vortexed and filtered (0.45 μm cellulose acetate). For quantification, 500 μL filtrate was mixed with 1 mL anthrone solution (2.0 g·L^−1^ in concentrated H_2_SO_4_), vortexed, cooled at 4 °C for 10 min, heated at 100 °C for 10 min, and cooled again in the dark for 10 min. An aliquot (200 µL) was transferred to a 96-well plate, and absorbance was measured at 620 nm.

### 2.8. Statistical Analysis

Statistical significance was determined by comparing protein, lipid, and carbohydrate levels between blank and test samples. Each group consisted of two biological replicates, each measured in technical triplicate, yielding 4–6 valid data points after excluding outliers. An unpaired *t*-test with Welch’s correction was performed using GraphPad Prism (version 8.0.2; GraphPad Software, San Diego, CA, USA) to account for unequal variances. One-way ANOVA with Tukey’s post hoc test was conducted in R (version 4.4.1) to confirm the robustness of the results; outcomes were consistent with those from the Welch’s *t*-test.

## 3. Results

### 3.1. Optimization of Biochemical Assays for Biomolecules in Soil

To validate the biochemical quantification assays, external standards were selected for each biomolecular class: bovine serum albumin (BSA) for proteins, soybean oil for lipids, and glucose for carbohydrates. Calibration curves were generated for each assay (Figure 2) and demonstrated strong linearity (R^2^ > 0.98), confirming their accuracy, precision, and reproducibility for quantifying microbial biomolecules in soil.

Protein content in soil samples was determined using the modified Lowry assay with BSA as the reference standard. The corresponding calibration curve (Figure 2a) exhibited excellent linearity (R^2^ > 0.99), confirming assay reliability. Lipid content was quantified using the SPV assay with soybean oil as the standard. The calibration curve (Figure 2b) showed strong linearity (R^2^ > 0.98), validating its suitability for lipid determination in soil. Carbohydrate content was determined using the anthrone assay with glucose as the standard. The calibration curve (Figure 2c) displayed excellent linearity (R^2^ > 0.98), ensuring precise carbohydrate quantification.

### 3.2. Biodegradation Experiments

**Figure 3:**
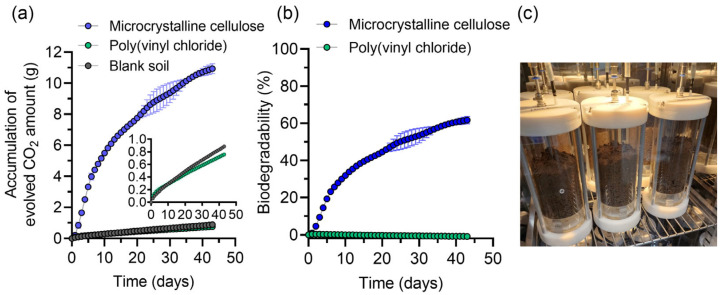
(**a**) Accumulation of evolved carbon dioxide (CO_2_) and (**b**) biodegradability in microcrystalline cellulose (MCC), poly(vinyl chloride) (PVC), and blank control vessels during 43 days of incubation. The inset in panel (**a**) shows a magnified view of the CO_2_ evolution in PVC and blank samples. (**c**) Experimental setup (photograph) shows the soil test vessels used for biodegradability assessment under controlled conditions (25 °C, aerobic, dark, 200 mL·min^−1^ airflow).

CO_2_ production in PVC was comparable to that in the blank control, indicating minimal microbial activity, whereas MCC exhibited a progressive increase in CO_2_ evolution, reaching 60% biodegradability by day 40 (Figure 3). This satisfies the ISO 17556:2019 validity criterion for the reference material (cellulose > 60% at plateau), confirming test validity. Variation between blank replicates was <20% (0.291 g and 0.294 g evolved), confirming experimental reproducibility.

### 3.3. Quantitative Analysis of Biomolecules in Soil

To link polymer biodegradability with microbial metabolism, soil protein, lipid, and carbohydrate contents were quantified at three time points: T_0_ (day 0, baseline), T_1_ (day 10, exponential phase; MCC = 33.72 ± 0.84% degraded), and T_2_ (day 40, plateau phase; MCC = 67.15 ± 1.98% degraded) using the optimized colorimetric assays (Figure 2).

At T_0_, baseline protein and carbohydrate concentrations did not differ significantly among treatments (*p* > 0.05), reflecting the indigenous biomass in the unsterilized soil. For example, the protein means for the blank and MCC soils were similar at 1289 µg g^−1^ soil (SD = 190.2 and 222.2, respectively; raw data in Table S2). Baseline lipids were modestly but significantly higher in the PVC (34.2 ± 7.5 µg g^−1^) and MCC (32.9 ± 11.9 µg g^−1^) soils than in the blank (16.0 ± 8.5 µg g^−1^; *p* < 0.05; Table S2), representing an ≤18 µg g^−1^ offset: minor compared with the >100 µg g^−1^ increases observed during MCC degradation.

**Figure 4:**
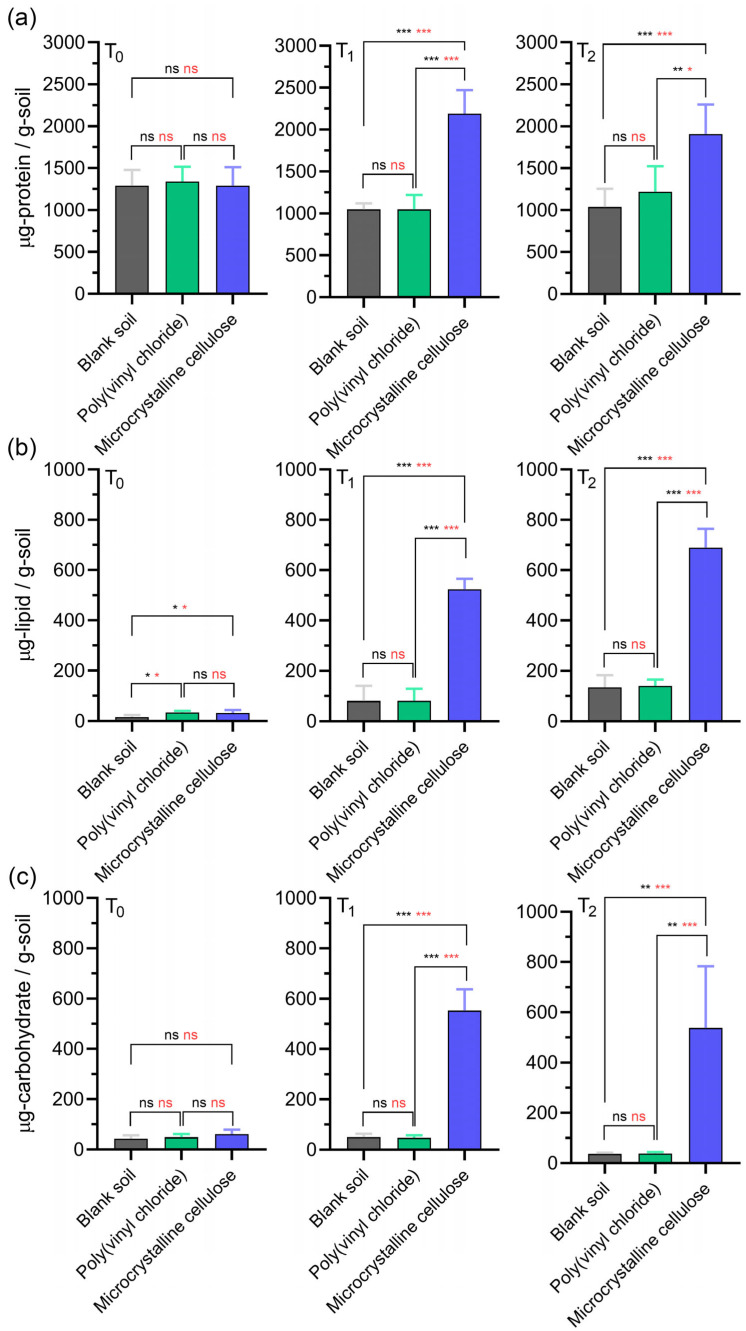
Quantitative analysis of (**a**) protein, (**b**) lipid, and (**c**) carbohydrate content in soil from blank, poly(vinyl chloride) (PVC), and microcrystalline cellulose (MCC) test vessels. Measurements were taken at T_0_ (day 0, initial phase), T_1_ (day 10, exponential phase), and T_2_ (day 40, plateau phase) using optimized colorimetric assays. Data are presented as mean ± standard deviation (*n* = 6 per treatment, two biological replicates × three technical replicates). Statistical significance was evaluated using two-tailed unpaired Welch’s *t*-tests (black asterisks) and one-way ANOVA followed by Tukey’s post hoc test (red asterisks). Significance levels: ns, not significant (*p* > 0.033); * *p* < 0.033; ** *p* < 0.002; *** *p* < 0.001.

By T_1_, MCC degradation coincided with sharp biomolecule enrichment: proteins, lipids, and carbohydrates increased by 2.09-, 6.47-, and 11.22-fold relative to the blank (all *p* < 0.001; Figure 4a–c). These increases paralleled the initial CO_2_ evolution burst (Figure 3), indicating active microbial growth on the cellulose substrate.

The stimulatory effect persisted until T_2_. Protein, lipid, and carbohydrate levels remained 1.84-, 5.13-, and 14.49-fold higher than the blank, respectively, even after CO_2_ evolution had plateaued (Figure 3), suggesting continued biomass accumulation beyond the point where carbon mineralization had leveled off.

In contrast, soils amended with PVC showed no statistically significant changes in any biomolecule over 40 days (all *p* > 0.05), underscoring the limited microbial response to this recalcitrant polymer.

**Figure 5:**
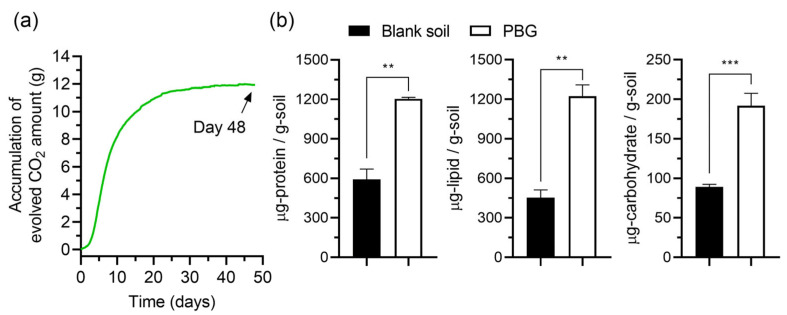
(**a**) Accumulation of evolved carbon dioxide (CO_2_) in poly(butylene glutarate) (PBG) and blank soil over 48 days of incubation. The arrow indicates the endpoint sampling at day 48. (**b**) Quantitative analysis of protein, lipid, and carbohydrate contents in soil collected on day 48 from blank and PBG vessels. Data are presented as mean ± standard deviation (*n* = 3 per treatment, three biological replicates). Statistical significance was evaluated using two-tailed unpaired Welch’s *t*-tests. Significance levels: *p* > 0.033); ** *p* < 0.002; *** *p* < 0.001.

A separate 48-day experiment with PBG corroborated the applicability of biomolecule quantification: PBG reached 70.1% biodegradation and induced 2.03-, 2.70-, and 2.15-fold increases in proteins, lipids, and carbohydrates, respectively (Figure 5), confirming that the assays captured microbial activation in response to degradable substrates.

## 4. Discussion

This study confirms the utility of biomolecular quantification as a complementary method for assessing polymer biodegradability in soil environments. For MCC, progressive CO_2_ evolution was accompanied by substantial increases in soil protein, lipid, and carbohydrate levels (Figure 3 and Figure 4), reflecting enhanced microbial activity during degradation. In contrast, PVC exhibited negligible changes in both CO_2_ production and biomolecule accumulation. A separate experiment using PBG further validated this approach, as PBG biodegradation was accompanied by measurable CO_2_ evolution and significant increases in proteins, lipids, and carbohydrates (Figure 5), consistent with previous findings on biodegradable polymer responses [22,23,24,25].

Statistical analyses, including Welch’s *t*-tests and one-way ANOVA with Tukey’s post hoc test, consistently revealed significant differences in biomolecule accumulation between biodegradable and non-biodegradable treatments. A minor but statistically significant baseline offset in lipid content was detected for PVC and MCC relative to the blank (≤18 µg g^−1^ soil; *p* < 0.05; Figure 4b). This small discrepancy, likely from trace lipidic additives or surface contaminants associated with the polymer powders, was an order of magnitude smaller than the lipid enrichment observed during MCC degradation and thus did not affect the overall interpretation.

The high linearity of protein, lipid, and carbohydrate calibration curves (Figure 2) underscores the reliability of these assays. DNA quantification was excluded because total DNA can persist after cell lysis and may not reflect active metabolism [32,33]. In contrast, proteins, lipids, and carbohydrates are functional biomarkers directly linked to microbial metabolic activity. Protein enrichment suggests microbial proliferation and enzymatic activity [34,35], lipid accumulation indicates sustained microbial growth and potential shifts toward lipid-rich taxa such as fungi or actinobacteria [36], and carbohydrate increases reflect enhanced exopolysaccharide production, which supports biofilm formation and substrate adhesion [37,38].

Importantly, high-molecular-weight MCC fragments were removed during centrifugation and filtration to ensure that measured carbohydrates originated from microbial metabolism rather than residual polymer [39]. The strong correlations between CO_2_ evolution and biomolecule accumulation observed for MCC and PBG highlight the capacity of this approach to capture microbial responses beyond carbon mineralization alone. While CO_2_ evolution effectively measures substrate mineralization, it may not fully represent biomass accumulation, enzymatic activity, or extracellular polymeric substances (EPS) production. Microbial growth can continue after CO_2_ production plateaus, particularly during the assimilation phase. Biomolecule quantification thus provides an additional layer of insight into microbial activity and degradation pathways [22,23,24,25].

Although analytically more demanding, this approach yields functional information that mineralization data alone cannot provide. This present study did not include scanning electron microscopy (SEM) or X-ray diffraction (XRD); Fourier-transform infrared spectroscopy (FTIR) was also not conducted. Additional limitations include the absence of abiotic degradation analyses [19,20], sterile controls (omitted in accordance with ISO 17556:2019 recommendations), and microbial community profiling. Future studies incorporating SEM, FTIR, and XRD, as well as metagenomics, transcriptomics, and enzymatic assays, could help distinguish biotic from abiotic degradation processes and identify the taxa and functions driving polymer biodegradation.

Despite these limitations, our results support biomolecular quantification as a sensitive and specific method for distinguishing biodegradable from non-biodegradable polymers. When combined with conventional CO_2_ and mass loss measurements, this approach can provide a more comprehensive understanding of polymer degradation mechanisms and microbial dynamics in environmental contexts.

## 5. Conclusions

This study demonstrates that biochemical quantification of microbial proteins, lipids, and carbohydrates provides a sensitive and reliable complement to conventional CO_2_-based assessments for evaluating polymer biodegradability in soil. The method effectively distinguished biodegradable from non-biodegradable polymers and showed strong concordance with CO_2_ evolution, while also revealing microbial physiological activity persisting beyond the mineralization plateau.

By extending assessment beyond mineralization and mass loss, biomolecule quantification captures additional aspects of microbial responses, such as biomass development and macromolecule accumulation, that CO_2_ measurements alone cannot resolve. This expanded perspective offers a more comprehensive understanding of microbial–polymer interactions in soil environments.

Future research should apply this approach to a wider variety of polymers, environmental conditions, and degradation scenarios. Integration with multi-omics techniques, including metagenomics and metabolomics, will further refine its applicability, enabling a deeper mechanistic understanding of the microbial and biochemical processes underpinning polymer biodegradation.

## Figures and Tables

**Table 1:** Preparation of test vessels for biodegradation experiments.

Constituent	Quantity	Description
Soil	500 g	Base medium for biodegradation experiments
KH_2_PO_4_	0.1 g	Essential minerals for microbial growth
MgSO_4_	0.05 g	Essential minerals for microbial growth
NaNO_3_	0.2 g	Nitrogen source for microbial growth
Urea	0.1 g	Additional nitrogen source
NH_4_Cl	0.2 g	Additional nitrogen source
Deionized H_2_O	60 mL	Added to achieve 55% soil water content

## Data Availability

All data are included in this article and in the Supplementary Materials.

## Supplementary Materials

The following supporting information can be downloaded at: https://www.mdpi.com/article/10.3390/polym17172376/s1, Figure S1: Photographs of the polymer samples tested in this study; Table S1: Molecular weight and thermal properties of the polymer samples; Tables S2–S4: Raw replicate data for biomolecule concentrations at T_0_, T_1_, and T_2_. References [40,41] are cited in the Supplementary Materials.
