# Supplementary material for: Refining Biodegradability Assessments of Polymers Through Microbial Biomolecule Quantification
Source: Polymers (Basel). 2025 Aug 31;17(17):2376. doi: 10.3390/polym17172376 (PMC12431410; doi:10.3390/polym17172376)
Supplement: Supplementary file 1 [file polymers-17-02376-s001.zip › polymers-3757086-supplementary.pdf]

Supplementary Materials

Refining Biodegradability Assessments of Polymers Through Microbial Biomolecule Quantification

Woo Yeon Cho and Pyung Cheon Lee

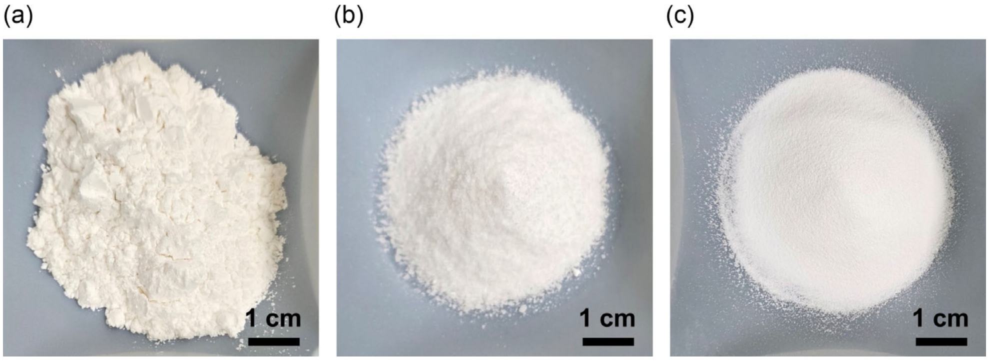

Figure S1. Photographs of the polymer samples tested in this study. (a) microcrystalline cellulose (MCC), (b) poly(butylene glutarate) (PBG), and (c) poly(vinyl chloride) (PVC). All images were taken under identical lighting and scale conditions. Scale bars represent 1 cm.

Table S1. Molecular weight and thermal properties of the polymer samples tested in this study: microcrystalline cellulose (MCC), poly(butylene glutarate) (PBG), and poly(vinyl chloride) (PVC). Reported parameters include weight-average molecular weight (M_w), dispersity (M_w/M_n), melting temperature (T_m), crystallization temperature (T_c), and glass transition temperature (T_g).

Polymer	M_w (kDa); M_w/M_n	T_m (°C)	T_c (°C)	T_g (°C)
Microcrystalline cellulose (MCC)	n.d.	n.d.	n.d.	132–184, multi-step T_g[a]
Poly(butylene glutarate) (PBG)	7.9; 1.5	42	- 12	- 62
Poly(vinyl chloride) (PVC)	116.6; 1.9[b]	n.d.	n.d.	n.d.

[a] Triple glass transitions (~132 °C, 159 °C, and 184 °C) have been reported for Avicel PH-102 (another commercial MCC grade) under dry conditions using modulated-temperature differential scanning calorimetry (MT-DSC) [1].

[b] Molecular weight (M_w) was determined by size-exclusion chromatography with multi-angle light scattering (SEC-MALS) for high-M_w PVC (Sigma-Aldrich 81387) [2].

M_w refer to weight-average molecular weight (kDa), M_n to number-average molecular weight (kDa), M_w/M_n to dispersity; T_g to glass transition temperature, T_m to melting temperature, T_c to crystallization temperature, and n.d. to not determined.

Table S2. Raw replicate data for biomolecule concentrations in soil at T_0 (day 0, initial phase) across treatment groups (Blank, PVC, MCC). Values are expressed as μg g^{-1} dry soil.

Biomolecule	Treatment	Rep 1	Rep 2	Rep 3	Rep 4	Rep 5	Rep 6	Mean ± SD
**Protein**	Blank	1169	1483	1479	1015	1200	1386	1289 ± 190.2
	PVC	1622	1266	1208	1307	1477	1157	1340 ± 176.5
	MCC	1280	1030	1403	1608	1046	1364	1289 ± 222.2
**Lipid**	Blank	13.48	13.79	28.18	8.56	-	-	16.00 ± 8.465
	PVC	36.67	31.67	22.35	40.83	39.32	-	34.17 ± 7.469
	MCC	25.23	35.08	53.18	31.89	34.39	17.65	32.90 ± 11.92
**Carbohydrate**	Blank	41.94	44.44	38.19	31.42	66.90	40.73	43.94 ± 12.09
	PVC	53.32	41.34	70.43	38.79	51.29	36.94	48.69 ± 12.58
	MCC	95.26	60.60	58.06	49.96	56.51	47.54	61.32 ± 17.35

Rep denotes replicate measurements consisting of two biological × three technical replicates (n = 6 per treatment where available). The symbol "–" indicates not measured. MCC refers to microcrystalline cellulose, PVC refers to poly(vinyl chloride) and T_0 (day 0, initial phase) is defined as in Figure 4.

Table S3. Raw replicate data for biomolecule concentrations in soil at T_1 (day 10, exponential phase) across treatment groups (Blank, PVC, MCC). Values are expressed as μg g^{-1} dry soil.

Biomolecule	Treatment	Rep 1	Rep 2	Rep 3	Rep 4	Rep 5	Rep 6	Mean ± SD
**Protein**	Blank	1118	1073	1116	922.7	1042	1015	1048 ± 73.50
	PVC	1104	766.4	930.5	1177	1227	1083	1048 ± 171.0
	MCC	2212	1884	2153	2165	2708	2015	2189 ± 281.2
**Lipid**	Blank	45.83	36.82	40.53	174.2	106.5	-	80.77 ± 59.49
	PVC	56.52	43.33	57.73	73.48	174.5	80.83	81.06 ± 47.65
	MCC	551.1	456.7	503.3	570.5	502.5	553.0	522.9 ± 42.76
**Carbohydrate**	Blank	51.51	34.61	30.26	56.42	60.00	62.93	49.29 ± 13.67
	PVC	44.78	34.18	38.97	62.89	50.47	50.26	46.93 ± 10.09
	MCC	485.0	473.8	468.2	629.7	625.3	635.3	552.9 ± 84.80

Rep denotes replicate measurements consisting of two biological × three technical replicates (n = 6 per treatment where available). The symbol "–" indicates not measured. MCC refers to microcrystalline cellulose, PVC refers to poly(vinyl chloride) and T_1 (day 10, exponential phase) is defined as in Figure 4.

Table S4. Raw replicate data for biomolecule concentrations in soil at T_2 (day 40, plateau phase) across treatment groups (Blank, PVC, MCC). Values are expressed as μg g^{-1} dry soil.

Biomolecule	Treatment	Rep 1	Rep 2	Rep 3	Rep 4	Rep 5	Rep 6	Mean ± SD
**Protein**	Blank	1335	1124	1184	750.8	862.1	975.4	1039 ± 216.4
	PVC	1561	1548	1354	916.8	950.0	975.4	1218 ± 305.5
	MCC	2120	2132	2054	1452	1472	2222	1908 ± 350.1
**Lipid**	Blank	104.3	175.0	153.6	200.0	77.12	96.14	134.4 ± 48.93
	PVC	142.6	140.6	176.6	150.8	132.7	98.64	140.3 ± 25.40
	MCC	676.7	608.6	603.3	734.1	793.3	723.1	689.8 ± 74.89
**Carbohydrate**	Blank	45.26	33.41	41.72	37.11	32.41	32.93	37.14 ± 5.309
	PVC	28.92	38.19	48.62	38.97	39.14	38.36	38.70 ± 6.240
	MCC	314.5	327.2	308.5	776.8	705.5	797.4	538.3 ± 244.7

Rep denotes replicate measurements consisting of two biological × three technical replicates (n = 6 per treatment where available). The symbol "–" indicates not measured. MCC refers to microcrystalline cellulose, PVC refers to poly(vinyl chloride) and T_2 (day 40, plateau phase) is defined as in Figure 4

References

1. Picker, K.M.; Hoag, S.W. Characterization of the Thermal Properties of Microcrystalline Cellulose by Modulated Temperature Differential Scanning Calorimetry. *J. Pharm. Sci.* **2002**, *91*, 342–349. https://doi.org/10.1002/jps.10028
2. Stepnov, A.A.; Lopez-Tavera, E.; Klauer, R.; Lincoln, C.L.; Chowreddy, R.R.; Beckham, G.T.; Eijsink, V.G.H.; Solomon, K.; Blenner, M.; Vaaje-Kolstad, G. Revisiting the Activity of Two Poly(Vinyl Chloride)- and Polyethylene-Degrading Enzymes. *Nat. Commun.* **2024**, *15*, 8501. https://doi.org/10.1038/s41467-024-52665-z
